# A Case Series on Successful Surgical Management of Patients Presenting With Lutembacher’s Syndrome to a Tertiary Healthcare Center in Northern India

**DOI:** 10.7759/cureus.44371

**Published:** 2023-08-30

**Authors:** Rajeshwar Yadav, Aditya Sharma, Swati Pathak

**Affiliations:** 1 Department of Cardiothoracic & Vascular Surgery, Institute of Medical Sciences, Banaras Hindu University, Varanasi, IND; 2 Department of General Surgery, Institute of Medical Sciences, Banaras Hindu University, Varanasi, IND

**Keywords:** lutembacher's syndrome, atrial septal defect, tricuspid annuloplasty, mitral valve replacement, mitral valve stenosis, atrial septal defect (asd), lutembacher syndrome (ls)

## Abstract

Atrial septal defect (ASD), whether congenital or iatrogenic, and mitral stenosis (MS), whether congenital or acquired, may all come together into a condition called Lutembacher’s syndrome (LS). The ASD is typically larger than 15 mm in a normal LS case.

However, congenital ASD is less frequent than residual iatrogenic ASD in the current era of percutaneous balloon mitral valvuloplasty for acquired MS. This is referred to as iatrogenic LS by cardiologists.

Hereby, we report a case series of three patients who presented to us, were diagnosed with LS, a very rare entity, and were managed successfully.

## Introduction

Johann Friedrich Meckel, an anatomist, first wrote about Lutembacher’s syndrome (LS) in 1750 [1]. Since then, various alterations have been made to the definition of LS. The lesions that should be included in the syndrome are a matter of debate. Some writers identify atrial septal defect (ASD) with mitral regurgitation (MR) as a subset of the LS spectrum, despite the fact that it is characterized as mitral stenosis (MS) in conjunction with ASD [2].

However, the current definition of LS includes any case of MS or ASD, regardless of whether it was congenital or acquired [3]. Both of the LS lesions could be congenital or acquired. Congenital ASD is a possibility in 0.6% to 0.7% of MS patients [4]. It is more likely to be common in regions where rheumatic heart disease is more common; however, the exact prevalence is not known [5].

The hemodynamic effects of this syndrome are a result of the interplay between the relative effects of ASD and MS. In the early stages of LS, it is believed that high left atrial pressure brought on by MS stretches open the patent foramen ovale, resulting in a left-to-right shunt and giving the left atrium another outlet.

In MS, this left-to-right shunt decompresses the left atrium, which causes the right ventricle to enlarge and experience pressure and volume overload, resulting in pulmonary artery hypertension (PAH). If severe PAH goes undiagnosed for a long time, a right-to-left shunt may form, worsening symptoms [6].

The duration or frequency of the symptoms can vary because most patients are initially asymptomatic, and certain symptoms may not manifest until later in life. The symptoms are often severe, including palpitations, ventricular overload, heart failure, and pulmonary congestion, which may be sudden and infrequent.

These symptoms may be less common, lasting only a few seconds, minutes, or even months, for symptoms such as right upper quadrant discomfort and ascites, as well as a loud mitral S1, pulmonary S2, mid-diastolic murmur, weariness, decreased exercise tolerance, weight gain, pedal edema, and lethargy.

We are describing this case series on LS, its presentation, and the treatment modalities available depending on the underlying cause of LS. There are a variety of surgical procedures available, which include closure of the ASD with pericardial patch, mitral valve replacement, and DeVega's annuloplasty for the tricuspid valve (TV) repair.

## Case presentation

A description of the various features of cases of LS diagnosed, admitted, and managed successfully by the mentioned procedures has been listed in Table 1.

**Table 1:** Description of the various features of cases of Lutembacher's syndrome diagnosed, admitted, and managed successfully by the mentioned procedures. MS: mitral stenosis; TR: tricuspid regurgitation; OS-ASD: ostium secundum-atrial septal defect; MVR: mitral valve replacement.

	Case 1	Case 2	Case 3
Age	46 years	42 years	32 years
Sex	Female	Male	Male
Symptoms	Breathlessness and fatigue	Breathlessness and dyspnea on exertion	Breathlessness and palpitations
Physical examination	Raised jugular venous pressure and mid-diastolic murmur	Raised jugular venous pressure and mid-diastolic murmur	Raised jugular venous pressure and mid-diastolic murmur
ECG	Tall peaked 'T' waves and 'QRS' right axis deviation	Tall peaked 'T' waves and 'QRS' right axis deviation	Tall peaked 'T' waves and 'QRS' right axis deviation
Pre-operative 2D echocardiogram	MS, TR, OS-ASD	MS, severe TR, OS-ASD	Moderate MS, moderately severe TR, OS-ASD
Coronary angiography	Normal coronaries	Not done	Normal coronaries
Surgical management	Closure of OS-ASD, MVR, DeVega's annuloplasty	Closure of OS-ASD, MVR, DeVega's annuloplasty	Closure of OS-ASD, MVR, DeVega's annuloplasty

Case 1

A 46-year-old married female with two live issues presented with the chief complaints of dyspnea for the past five years and easy fatigability for the previous six months. When she first experienced breathlessness five years ago, she was ostensibly asymptomatic. It came on gradually and insidiously; it was first increased by effort but relieved by resting, but in the last six months, it has also been linked to easy fatigue. Fever, cough with expectoration, night sweats, joint discomfort, or bluish discoloration of the skin, nails, or mucous membranes were not present in the past. There is no history of similar illnesses in the family, any comorbidities, or any surgical interventions in the past.

On general examination, the patient was averagely built and undernourished and had a body mass index (BMI) of 16 kg/m2. Her pulse rate was 108 beats per minute, hypovolemic and irregular, recorded in the right radial artery without any radio-radial or radio-femoral delay, and 118/72 mm Hg was the blood pressure with a respiratory rate of 22 cycles per minute (thoraco-abdominal).

Pallor and pedal edema were present, and jugular venous pressure (JVP) was raised, with a mean JVP of 2 cm above the sternal angle in a 45-degree head-up position; hepatojugular reflux was absent, with normal respiratory variation and no venous hum.

The chest was bilaterally symmetrical on cardiovascular system examination, with an apex impulse apparent on the sixth intercostal space (ICS), and there were no other scars, sinuses, or dilated veins. An apical impulse was palpable just lateral to the midclavicular line in the sixth ICS. The left cardiac boundary corresponded to the apex.

A loud S1 and a wide, fixed, split S2 were audible on auscultation. A grade 1 mid-diastolic murmur was noted at the mitral area that was localized, not radiating to any other sites, low pitched, rumbling in nature, and best audible with a bell positioned to the left of the heart; it increased with expiration, decreased with inspiration, and increased with standing (MS). A pansystolic murmur was best heard with the diaphragm at the tricuspid area. It was grade 2 at the left lower parasternal region, which was high-pitch blowing and plateau in configuration radiating to the apex (tricuspid regurgitation).

In this patient, the left 2nd ICS in the parasternal area was the ideal place to listen for a grade 2 ejection systolic murmur. It was a brief, crescendo-decrescendo, medium-frequency, early peaking murmur that is best detected with the diaphragm near the end of the expiration.

During the assessment of the respiratory system, only the typical vesicular breath sounds, bilaterally equal, were audible. An examination of the abdomen revealed a soft abdomen, a slight pain in the right hypochondrium, and moderate hepatomegaly.

**Figure 1:**
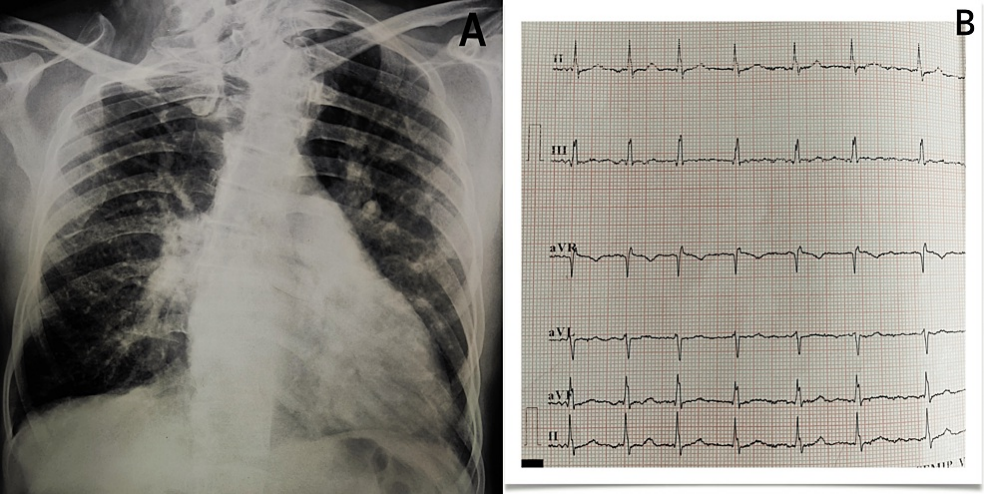
(A) A chest X-ray showing the left pulmonary artery is prominent, there is cardiomegaly, primarily enlargement of the right ventricle, and the left heart border has straightened. (B) An electrocardiogram (ECG) showing tall and peaked T waves along with QRS right axis deviation in Case 1.

The patient underwent various sets of investigations following a thorough physical and systemic assessment. Hematological values fell within acceptable ranges. On a chest X-ray, the left pulmonary artery was prominent, there was cardiomegaly, primarily enlargement of the right ventricle, and the left heart border had straightened. On an electrocardiogram (ECG), tall, peaked T waves and a right-axis deviation of the QRS were also visible, as shown in Figure 1.

The transthoracic two-dimensional (2D) echocardiography revealed moderate PAH and pulmonary artery systolic pressure (PASP) of 50 mmHg from the tricuspid regurgitation (TR) jet, both indicators of severe TR. According to the patient's clinical presentation, the patient was diagnosed with significant MS and a wide ostium secundum-atrial septal defect (OS-ASD). These findings were consistent with LS.

Before undergoing surgery that could have a significant impact on the patient, proper preparation and optimization were carried out because the patient arrived at our facility in poor general health and with congestive heart failure. As diuretics and supportive therapy were used to treat the patient's congestive failure, she made a significant recovery. The patient was scheduled for elective surgery after receiving adequate decongestive treatment.

A temperature probe, a radial artery line, central and peripheral venous lines, and bispectral index (BSI) monitoring were all started on the table. Fentanyl 0.5 mcg/kg and etomidate 0.2 mg/kg were administered intravenously to induce anesthesia. Using a 7-mm ID endotracheal (ET) tube and 0.1 mg/kg of vecuronium, the tracheal intubation was aided. The left ventricle was tiny in size, while the right atrium (RA), right ventricle, and left atrium were all greatly enlarged.

The appropriate atriotomy was performed following a proper assessment and time on the pump. The anterior mitral leaflet (AML) was removed while leaving the sub-valvular system intact. A 27-mm St. Jude's bi-leaflet metallic valve and Ethibond 2-0 sutures were used to replace the mitral valve (MV). An autologous pericardial patch made of prolene 4-0 was used to close the ASD. The ASD sutures were placed after de-airing the left side of the heart.

DeVega’s TV annuloplasty was then done using prolene 2-0 sutures. After rewarming was begun, RA closure was done simultaneously. After de-airing was completed, the patient was taken off the pump. Decannulation proceeded as the heart ejections became regular and good contractions and vital parameters were visible. The right-side heart's size significantly decreased right away after excellent ejections. Once good hemostasis was obtained, the sternum was closed. In stable condition, the patient was transferred to postoperative care.

The patient was ventilated all night to help the heart and lungs adjust gradually and without discomfort to the new hemodynamic flows. There was very little inotropic support used postoperatively. During the postoperative period, the patient did not experience any cardiac rhythm events. Apart from good antibiotic and diuretic therapy, anticoagulants were begun.

Along with international normalized ratio (INR) monitoring, injections of enoxaparin (0.6 mg) and oral warfarin (2 mg) were started on postoperative day (POD) one. One by one, mediastinal drains were removed by POD three. After an uneventful hospital stay, the patient was ambulatory on POD three and was discharged on oral medications on POD 10.

The patient was frequently followed up after discharge, first after 15 days and then two months later. Remodeling began magically bringing the heart size to normal levels over time. The target INR was kept between 2.5 and 3.5 by routinely monitoring the INR and adjusting the warfarin dose accordingly.

The results of the subsequent echocardiography were consistent with an in situ prosthetic MV with normal function and no MR or paravalvular leak. The maximum pressure gradient was 8 mm Hg and the mean pressure gradient was 5 mm Hg, with left ventricular ejection fraction (LVEF) of 50-55% and mild TR, and right ventricular systolic pressure (RVSP) of 16 mm Hg. There was no flow across the interatrial septum during the study. No clot or vegetation was noted.

Case 2

A 42-year-old male presented with chief complaints of breathlessness from the past eight years associated with dyspnea on exertion from the past two years. He was reportedly asymptomatic eight years ago when he mysteriously started to have breathlessness that worsened with activity but subsided with rest. For the past two years, however, it has also been linked to dyspnea when exerting oneself. There is no family history of the illness, no concomitant conditions, and no prior surgical procedures.

Upon general examination, the patient was underweight and had a BMI of 14.8 kg/m2. His pulse rate was erratic and hypovolemic at 98 beats per minute, and his blood pressure was 102/66 mm Hg. The respiratory pattern was thoraco-abdominal, and the rate was 18 cycles per minute. The mean JVP was elevated 2 cm above the sternal angle. An apex impulse was seen in the sixth ICS during a cardiovascular system examination. During auscultation, a loud S1 and a wide-spilt S2 were audible, along with a grade 1 mid-diastolic murmur at the mitral region.

During the respiratory system assessment, normal vesicular breath sounds were audible on both sides equally, without any additional sounds. The abdomen was soft and non-tender, and further palpation revealed no signs of organomegaly. The hematological variables fell within acceptable bounds.

On a chest X-ray, cardiomegaly is mostly seen as right ventricular enlargement and left heart border straightening. The right axis deviation of the QRS and tall peaked T waves were also visible on the ECG. Acyanotic chronic heart disease with a bigger OS-ASD (size 33-34 mm; noticeably deficient superior rim) and left to right atrial shunt with a Qp/Qs ratio of 4.9:1 was revealed on transthoracic 2D echocardiography along with the other findings of severe TR, right-sided volume overload, dilated main pulmonary artery (MPA) with moderately severe TR, moderate to severe (hyperkinetic) PAH, rheumatic involvement of MV, and mild to moderate MS.

**Figure 2:**
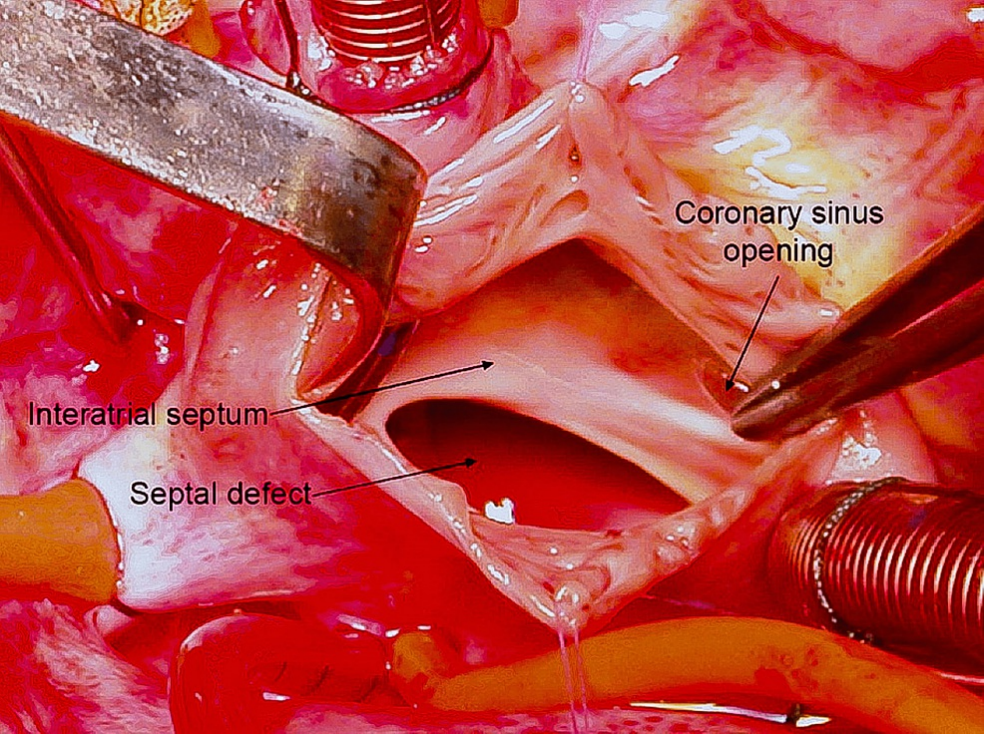
An intraoperative picture showing a large atrial septal defect and coronary sinus opening along with inter-atrial septum in Case 2.

After stabilization, the patient was scheduled for the closure of the large ASD and intraoperatively a large ASD was noted as seen in Figure 2.

Alongside MV replacement, TV annuloplasty was also done successfully. He was subsequently transferred to postoperative care, where the postoperative time went smoothly. The patient was released on POD 10 and fared well throughout his follow-up visits.

Case 3

A 32-year-old male presented to us with chief complaints of breathlessness for the past three years, associated with palpitations for the past two months. When he first had breathlessness three years ago, it was seemingly asymptomatic and gradual in nature. It first worsened with exercise but subsided with rest, but for the past two months, it has also been accompanied by palpitations. There is no history of similar illnesses in the family, any comorbidities, or any surgical interventions in the past.

On general examination, the patient was averagely built with a BMI of 17.8 Kg/m2. He was afebrile with a pulse rate of 88 beats per minute. He had a blood pressure of 134/82 mm Hg with a respiratory rate of 16 cycles per minute, and JVP was raised.

During the cardiovascular system exam, the chest was bilaterally symmetrical with an apical impulse apparent on the sixth ICS; no other scars, sinuses, or dilated veins were found. All of the inspection's conclusions were verified upon palpation. In conjunction with grade II left parasternal heave, a diffuse apical impulse was noticed in the 6th ICS, just lateral to the midclavicular line. The boundary of the left heart corresponded to the apex during the percussion.

A loud S1 and a wide, fixed, split S2 were audible on auscultation. At the mitral region, a grade 1 mid-diastolic murmur could be noted. The left 2nd ICS in the parasternal area is where an ejection systolic murmur of grade 2 is most often detected. During the respiratory system examination, bilaterally equal normal vesicular breath sounds were heard with no added sounds. On per abdomen examination, the abdomen was soft and non-tender, and there was no evidence of organomegaly on palpation. The central nervous system examination was within normal limits.

The hematological parameters were within normal limits. On chest X-ray, cardiomegaly is predominantly right ventricular enlargement. The ECG was showing tall and peaked T waves along with QRS right axis deviation. The transthoracic 2D echocardiography revealed acyanotic chronic heart disease with larger OS-ASD, moderately severe TR, moderate to severe (hyperkinetic) PAH, and moderate to severe MS.

**Figure 3:**
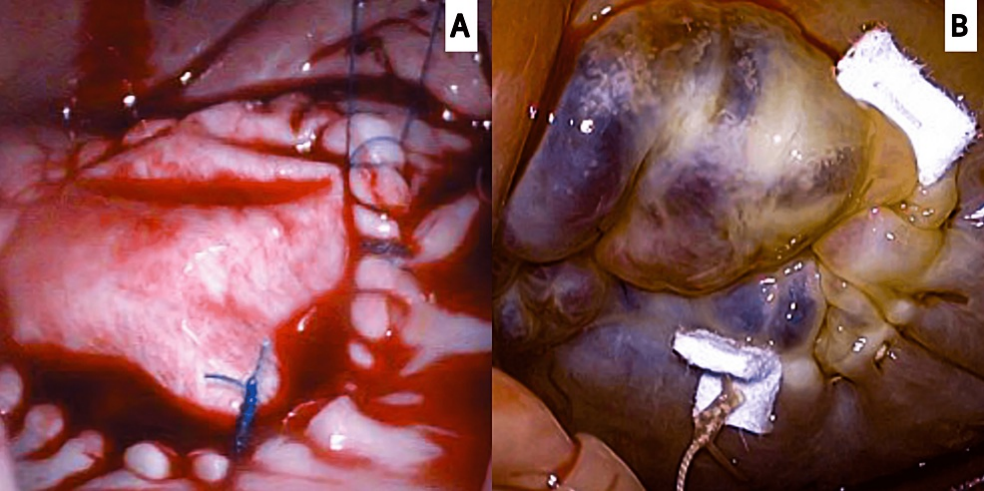
(A) An intraoperative picture showing the successful closure of an atrial septal defect with a pericardial patch using prolene 4-0 sutures. (B) Another intraoperative picture showing DeVega’s tricuspid valve annuloplasty done using prolene 2-0 sutures.

The patient was planned for the closure of ASD, MV replacement, and TV annuloplasty, as shown in Figure 3.

After stabilization, the patient was shifted to postoperative care. The postoperative period was uneventful. He was discharged on POD 13, and the patient did well during his follow-up visits.

## Discussion

According to René Lutembacher (1916), "classical Lutembacher's syndrome" is a congenital ASD compounded by acquired MS [7]. Although Corvisart initially noted an association between ASD and MS in 1811, in Lutembacher's original report from 1916, when the patient was a 61-year-old woman who had given birth seven times, the ameliorating effect of the ASD on MS was clear. This condition very rarely occurs [8]. According to a 1997 study that appeared in the American Heart Journal, the prevalence of Lutembacher's condition was 0.001 per 100,000 people [9].

In LS, opening snaps and presystolic accentuation are less commonly observed. Additionally, the auscultatory characteristics of MS are missed as a result of the left ventricle's posterior displacement. When there is a significant ASD and a high left atrial pressure due to MS, irreversible pulmonary vascular disease is extremely rare. Our current understanding of this illness allows us to say that both ASD and MS can be either acquired or congenital. The use of echocardiography aids in separating the typical form of LS from iatrogenic LS [10].

In typical LS ASD, the diameter is large and non-restrictive, but the diameter ranges from 0.5 to 1.0 cm (restrictive) in iatrogenic LS ASD. In the mentioned three cases, there was a large ASD, so it masked the signs and symptoms of MS as it reduced the gradient across the mitral valve, resulting in a decrease in the intensity of the murmur.

The diagnosis of LS is made by transthoracic echocardiogram (TTE), as it helps identify the type and size of ASD and the severity of MS. In classical LS, the ASD should be greater than 1.5 cm^2^. The subcostal window is the preferred view to visualize ASD over the apical four-chamber view, in which echo dropouts are a problem [11].

Perloff cited an earlier case report from 1880 that described a 74-year-old woman who had experienced 11 pregnancies. A favorable prognostic value is aided by early diagnosis and surgical intervention [12]. The prognosis is poor if the patient is detected too late and develops heart failure and pulmonary hypertension.

ASD closure with mitral valve replacement provides an excellent prognosis and prolongs survival if the patient is diagnosed earlier, before the onset of pulmonary hypertension and heart failure.

## Conclusions

LS is a rare congenital cardiac condition that presents in adults. The interplay between the hemodynamics caused by ASD and MS determines the presentation and outcome (often leading to a missed diagnosis of MS). Cardiomegaly, responsible for rhythm disturbances, aggravates the challenge of operating on dilated hearts. Multiple repair procedures and replacements may be required (TV annuloplasty). The heart undergoes major hemodynamic changes and remodeling after the operative correction. In rare conditions, proper evaluation is necessary before considering operative correction.
